# Sex-Specific Changes and Correlates of Intimate Partner Violence in Kenya: Analyses of Nationally Representative Surveys From 2014 and 2022

**DOI:** 10.1177/08862605261444013

**Published:** 2026-05-30

**Authors:** Anne Abio, Mahsa MohammadNamdar, Omid Dadras, Michael Lowery Wilson, Jacinta Mukulu Waila

**Affiliations:** 1 Research Centre for Child Psychiatry, University of Turku, Finland; 2 INVEST Research Flagship Center, University of Turku, Finland; 3 Injury Epidemiology and Prevention (IEP) Research Group, Turku Brain Injury Centre, Department of Clinical Neurosciences, Turku University Hospital and University of Turku, Finland; 4 Heidelberg Institute of Global Health (HIGH), University Hospital and University of Heidelberg, Germany

**Keywords:** physical violence, psychological violence, sexual violence, sub Saharan Africa, cross-sectional survey, demographic health survey

## Abstract

Intimate partner violence (IPV) impacts both females and males with long-lasting consequences. The study investigated changes in IPV, co-occurrence of multiple forms of violence, and factors associated with IPV among females and males in Kenya. We used secondary data from the 2014 and 2022 Kenyan Demographic Health Surveys. Logistic regression analysis was used to estimate changes in IPV and the association with explanatory factors stratified by sex. Among females, lifetime prevalence of any IPV was stable in 2014 versus 2022 (47.1% vs. 46.3%; *OR* = 1.09, 95% CI [0.98, 1.22]), and in the past year (32.6% vs. 31.1%; *OR* = 1.05, 95% CI [0.94, 1.18]). Psychological IPV among women increased modestly (lifetime *OR* = 1.31, 95% CI [1.17, 1.47]; 12-month *OR* = 1.18, 95% CI [1.04, 1.34]). Among males, lifetime prevalence of any IPV rose from 23.4% to 29.7% (*OR* = 1.78, 95% CI [1.50, 2.12]), psychological IPV from 20.4% to 26.5% (*OR* = 1.77, 95% CI [1.48, 2.12]), and sexual IPV from 3.8% to 4.9% (*OR* = 1.68, 95% CI [1.16, 2.45]). Past‑12‑month any IPV (*OR* = 1.38, 95% CI [1.14, 1.66]) and psychological IPV (*OR* = 1.43, 95% CI [1.18, 1.73]) also increased. Across both sexes, older age, father beat mother, and perpetration of physical violence were associated with higher odds of IPV, while higher education was associated with lower odds of IPV among women but higher odds among men. Lifetime co-occurrence of three forms of IPV were reported at 8.5% versus 7.9% in women, and 1.4 versus 1.1% in men (2014 vs. 2022). While overall IPV among women remained unchanged in 2014 and 2022, psychological violence rose, and men reported significant increases in both lifetime and recent IPV. Distinct sex-specific patterns in associated factors underscore the need for sex-responsive and inclusive IPV prevention strategies, particularly interventions addressing psychological abuse and intergenerational violence.

## Introduction

Intimate partner violence (IPV) is a widespread public health issue with serious consequences for individuals, families, and communities. IPV refers to any form of physical, psychological, sexual, or financial abuse inflicted by a current or former partner in an intimate relationship (Krug et al., 2002; Oram et al., 2022). It impacts individuals across all races, social classes, ages, socioeconomic backgrounds, and genders (Renner & Whitney, 2012), although its manifestation varies. Globally, IPV affects millions of females and males, and has severe implications for mental and physical health, including long-term social and economic costs (Jonas et al., 2011; Rhys et al., 2019). In sub-Saharan Africa, including Kenya, IPV remains a critical challenge. During the pandemic, roughly 21% to 31% of women reported experiencing any form of IPV, while studies before COVID-19 reported 27% to 30% for physical and/or sexual violence (Costa et al., 2024; Ghahramani et al., 2024; Kifle et al., 2024; Sardinha et al., 2022; World Health Organization, 2013, 2021).

Research has consistently shown that women are disproportionately affected by IPV, with the types of violence often varying by region, socioeconomic background, and access to support services (Costa et al., 2024; Ghahramani et al., 2024; Kifle et al., 2024; Sardinha et al., 2022). In Kenya, the lifetime prevalence of IPV against women ranges from 20% to 78%, depending on the characteristics of women being studied (Memiah et al., 2021; Stiller et al., 2022). Moreover, nationally representative data revealed that 4 out of 10 women in Kenya reported exposure to IPV in 2022 (Kenya National Bureau of Statistics et al., 2023) despite concerted efforts to address IPV through legal frameworks and awareness programs by the Kenyan Government.

### Legal Framework and the COVID-19 Pandemic

The Kenyan Government recognizes IPV violence as a problem, and as such has a national policy for the prevention of gender-based violence (National Policy for Prevention and Response to Gender-Based Violence, 2014). Additional frameworks include the Penal Code, the Constitution of Kenya, the Criminal Procedure Code, and the Sexual Offenses Act; however, effective implementation of the laws are hampered by inadequate enforcement of laws and resources (National Policy for Prevention and Response to Gender-Based Violence, 2014). In response to COVID-19, curfews and school closures were enforced as mitigation measures (Reed et al., 2023). The COVID-19 pandemic exacerbated experiencing IPV due to various factors, such as economic stress/shock, increased substance and alcohol use, and reduced access to support systems (Costa et al., 2024; Reed et al., 2023; Uzoho et al., 2023). These factors emphasize the importance of providing evidence-based estimates about IPV for both sexes.

### Conceptual Framework

This study conceptualizes IPV as rooted in gendered power relations, social norms, and the intergenerational continuation of violence (Jewkes et al., 2015; National Policy for Prevention and Response to Gender-Based Violence, 2014). The COVID-19 pandemic is treated as a shock that may have intensified gender inequalities and relationship stress, thereby influencing IPV risk in 2022. Individual variables such as age, place of residence, education level, perpetration of physical violence against a partner, and parental violence are avenues through which gender norms and power relations are exerted (Das et al., 2013; Gibbs et al., 2020). Education and residence may reflect differential access to resources, while parental violence represents intergenerational normalization of violence. Perpetration of violence is conceptualized as an expression of gendered power and a marker of reciprocal or co-occurring IPV dynamics (Jewkes et al., 2015). Sex-disaggregated analyses enable examination of how these factors and how their associations and, co-occurrence of different forms of IPV changed in Kenya before and during the pandemic.

### Rationale for the Study

There is a notable gap in understanding the magnitude of IPV among males in low and middle-income countries, as well as how these patterns might differ over time (Jud et al., 2023). Studies have suggested that IPV against men is underreported or less recognized since support is more often offered to women, while men are referred to law enforcement (Perryman & Appleton, 2016; Powney & Graham-Kevan, 2019). Although there is a wealth of literature on female IPV victimization, mixed findings were reported regarding the experience of IPV during the COVID-19 pandemic (Uzoho et al., 2023), underscoring the need to estimate if IPV changed in Kenya before and during the pandemic. This study aims to: (a) assess the changes in the prevalence of reported IPV and co-occurrence of different forms of violence, and (b) assess the factors associated with IPV among females and males in Kenya in 2014 and 2022 using a nationally representative sample.

## Methodology

### Sampling Procedures

The study used data from Kenyan Demographic Health surveys (KDHS) conducted in 2014 and 2022. These surveys constituted the 6th and 7th KDHS in the country. The data collection on domestic violence among men was introduced in 2014 in the Kenyan DHS. Data collection took place from May to October 2014 and from February to July 2022. The surveys provide nationally representative and country level information on sociodemographic, nutrition, and health indicators. The country is divided into 47 counties, and these were stratified into urban and rural strata. A two-stage sampling strategy was used, and clusters were selected independently of each sampling stratum. In about three quarters of the sample, women were interviewed about the domestic violence module while men were targeted in a quarter of the sample.

### Study Population

A total of currently or ever married/cohabiting 5,657 women and 4,962 men in 2014; and 16,926 women and 5,683 men in 2022 were interviewed for the domestic violence module. The analytic sample was restricted to a total of 4,519 women and 3,289 men in 2014; and 12,888 women and 3,614 men in 2022 who responded to the intimate partner questions. Permission to use the data was sought from the DHS team. Participation in the survey was voluntary, and the data provided to the researchers was de-identified; thus, no additional ethical approval was obtained for this study. Additional information about the survey, methodology, and ethical approval is available from the DHS website (Kenya National Bureau of Statistics et al., 2015, 2023).

### Variables

The main outcome was the experience of IPV reported among currently or ever-married or cohabiting couples. This included the lifetime experience or experience in the 12 months prior to the survey among males and females. IPV was categorized into physical violence, psychological violence, and sexual violence. To determine the experience of physical violence, participants were asked if their partner had pushed, shaken, thrown something, twisted their arm, pulled their hair, punched, kicked, dragged, beaten, choked, burned, or attacked them with a knife, gun, or other weapon. Psychological violence was determined by asking participants if their partner had said or done something to humiliate them in front of others, threatened to hurt them or someone they cared about, or insulted them to make them feel bad about themselves. Sexual violence was determined by asking if participants’ partners had physically forced them to have sexual intercourse or perform any sexual acts they did not want, including using threats. The responses to these questions were no or yes (often, sometimes, not in the last 12 months) and coded as binary – *no* and *yes*. This was used to determine the experience of IPV during their lifetime or in the 12 months preceding the survey. Explanatory variables included year of data collection (coded as 2014 and 2022), age (categorized 15–24, 25–34, and 35–49 for females and 15–24, 25–34, 35–44, and 45–54 for males), place of residence (urban vs. rural), level of education (no education, primary, secondary, higher), parental violence through the respondents’ father beating their mother (no, yes, don’t know), and perpetrated physical violence against their partner (no vs. yes). The year of data collection was the main predictor variable with 2014 as the reference and compared to 2022.

### Data Analysis

The survey design of the data was taken into account during the analysis. Frequencies and percentages were estimated for categorical variables. The change in IPV between 2014 and 2022 was determined using Pearson chi-square tests and logistic regression. The analyses were stratified by sex considering that the datasets were provided separately by sex and included different domestic violence weight variables. The domestic violence weight variable was modified to adjust for the population by sex in 2014 and 2022. The association between IPV and the explanatory variables was estimated using binary logistic regression analysis. The adjusted models included age, place of residence, level of education, perpetrated physical violence against a partner, and father beat mother since they are associated with IPV in previous studies (Das et al., 2013; Gibbs et al., 2020; Stiller et al., 2022). Separate models were fitted for physical violence, psychological violence, and sexual violence for lifetime experience and 12 months prior to the survey. Year-specific regression analyses stratified by sex assessed if there were any changes in observed associations with IPV in 2014 and 2022. Statistical significance was set at *p* < .05 with a corresponding 95% confidence interval (CI). Data analyses were conducted using Stata 18 (StataCorp TX, United States).

## Results

### Females

During both time periods (2014 and 2022), there was no difference in the lifetime report of any form of IPV (47.1% vs. 46.3%) (Table 1). Psychological violence reported was higher in 2022 (32.4% vs. 36.3%), while physical violence was lower in 2022 (37.0% vs. 33.0%). Regarding the 12-month period prior to each survey, there was no difference in any IPV (32.6% vs. 31.1%) and psychological violence reported (23.8% vs. 24.9%) in 2014 and 2022, respectively. The 12-month physical violence and sexual violence reported were both lower in 2022. Furthermore, perpetration of physical violence against a partner was lower in 2022 (3.8% vs. 2.8%). The proportion of women who reported that their fathers beat their mothers was also lower in 2022 (37.6% vs. 32.5%). Supplemental Figure 1 illustrates the changes of IPV types for 2014 and 2022.

**Table 1.** Prevalence of IPV and Participant Background Characteristics.

Variable	Females					Males				
	2014		2022		*p*-value	2014		2022		*p*-value
	*N*	Weighted percent	*N*	Weighted Percent		*N*	Weighted percent	*N*	Weighted percent	
IPV, lifetime					.549					<.001
No	2,497	52.87	7,117	53.66		2,539	76.6	2,621	70.33	
Yes	2,022	47.13	5,771	46.34		750	23.4	993	29.67	
Psychological IPV, lifetime					.002					<.001
No	3,142	67.62	8,448	63.73		2,642	79.65	2,729	73.55	
Yes	1,373	32.38	4,440	36.27		642	20.35	885	26.45	
Physical IPV, lifetime					.002					.362
No	2,906	63.04	8,684	67.04		3,042	92.97	3,357	92.12	
Yes	1,609	36.96	4,204	32.96		243	7.03	257	7.88	
Sexual IPV, lifetime					.052					.131
No	3,985	86.72	11,584	88.4		3,162	96.21	3,458	95.06	
Yes	529	13.28	1304	11.6		123	3.79	156	4.94	
IPV, past 12 months					.215					.192
No	3,104	67.39	8,964	68.9		2,708	82.56	2,944	80.75	
Yes	1,411	32.61	3,924	31.1		556	17.44	670	19.25	
Psychological IPV, past 12 months					.320					.077
No	3,490	76.19	9,802	75.07		2,795	85.27	3,024	82.95	
Yes	1,025	23.81	3,086	24.93		468	14.73	590	17.05	
Physical IPV, past 12 months					<.001					.782
No	3,517	77.5	1,0498	81.75		3,098	95.13	3,446	94.92	
Yes	998	22.5	2,390	18.25		166	4.868	168	5.084	
Sexual IPV, past12 months					.005					.661
No	4,116	90.2	1,2016	92.29		3,166	96.88	3,507	96.58	
Yes	397	9.8	872	7.71		97	3.123	107	3.418	
Age					<.001					.122
15–24	892	18.86	2,306	16.21		201	6.105	216	6.453	
25–34	2,026	43.87	5,634	41.67		1,320	39.76	1,359	35.85	
35–44 (35–49)*	1,601	37.27	4,948	42.12		1,135	32.98	1,320	33.89	
45–54						633	21.15	719	23.81	
Place of residence					.898					.040
Urban	1,644	39.47	4,831	39.23		1,341	43.82	1,431	39.22	
Rural	2,875	60.53	8,057	60.77		1,948	56.18	2,183	60.78	
Education level					<.001					<.001
No education	788	9.33	2,038	7.27		260	3.888	284	4.008	
Primary	2,456	56.42	5,276	42.88		1,736	52.09	1,548	44.03	
Secondary	971	25.99	3,702	31.93		869	29.64	1,043	30.11	
Higher	304	8.26	1,872	17.92		424	14.38	739	21.86	
Perpetrated physical violence against partner					.038					<.001
No	4364	96.19	12,556	97.18		2,139	62.4	2,703	74.62	
Yes	151	3.81	332	2.82		1,126	37.6	911	25.38	
Father beat mother					<.001					<.001
No	2,505	56.4	8,196	62.5		1,493	44.67	1,825	51.89	
Yes	1,757	37.62	4,084	32.48		1,585	48.84	1,549	41.95	
Don’t know	250	5.98	608	5.02		181	6.488	240	6.158	

*Note. N* = unweighted count; IPV = Intimate partner violence.

* 35–44 for males and 35–49 for females.

**Figure 1.**
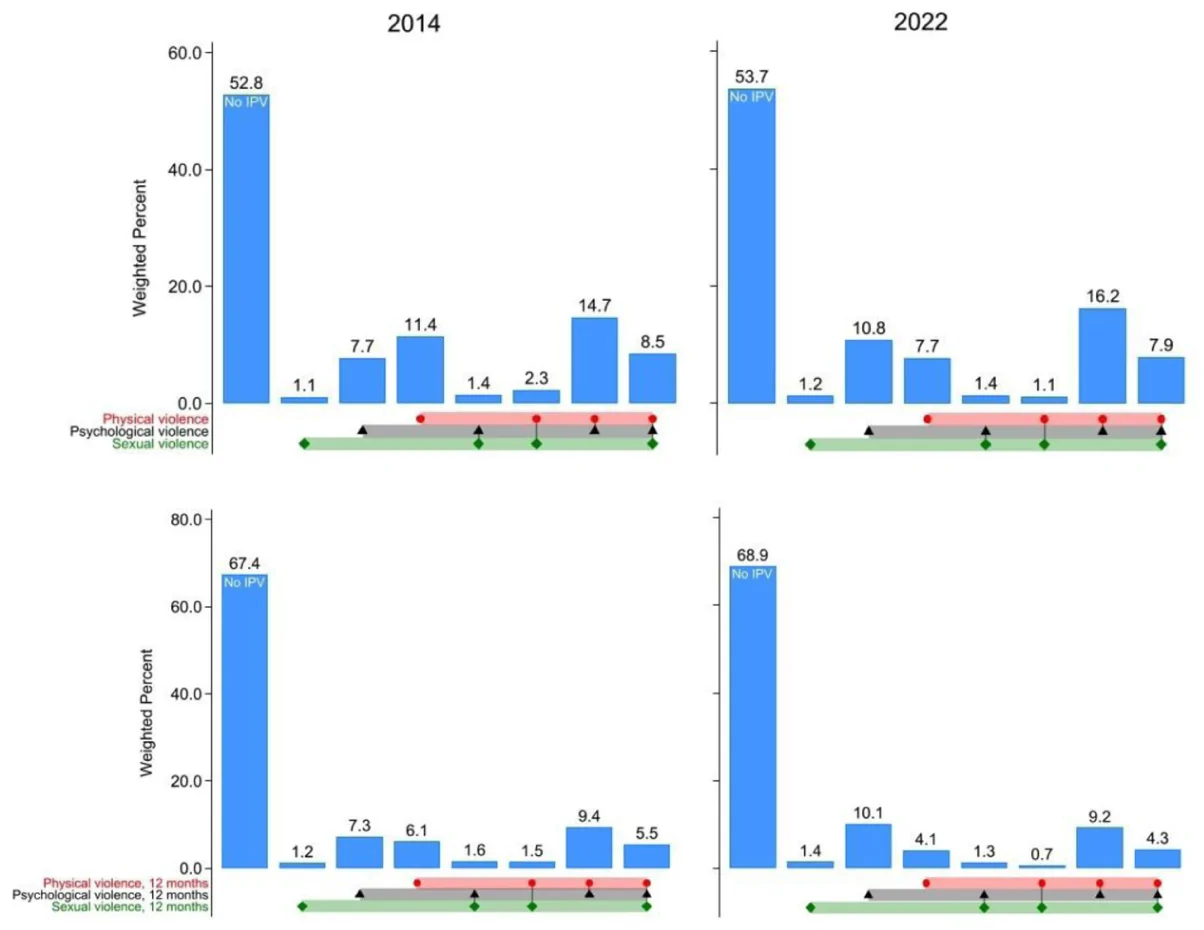
Profile of IPV types for females – Lifetime (above) and past 12 months (below). *Note.* Vertical bars represent the prevalence of respective combinations of types of IPV with non-missing numbers. IPV: intimate partner violence.

Regarding the lifetime reports of more than one form of violence, a combination of three forms of violence was 8.5% in 2014, and 7.9% in 2022 (Figure 1). The co-occurrence of both physical and psychological violence were the most frequently reported at 14.7% in 2014 and 16.2% in 2022. The least frequent combination reported was psychological with sexual violence in 2014 (1.4%), which changed to physical with sexual violence in 2022 at 1.1%. In the 12-month recall period, 5.5% in 2014 and 4.3% of females in 2022 reported all three forms of violence. Congruently, the most frequent combination reported was physical and psychological violence at 9.4% in 2014 and 9.2% in 2022. However, the least frequent combination was physical with sexual violence at 1.5% and 0.7% in 2014 and 2022, respectively.

Regression analyses revealed the lifetime odds of any form of IPV was 9% higher in 2022 (*OR* = 1.09, 95% CI [0.98, 1.22]), although marginally insignificant (Table 2a). Females were 31% more likely to report psychological violence in 2022 (*OR* = 1.31, 95% CI [1.17, 1.47]). Although not statistically significant, the odds of physical and sexual violence were lower in 2022. A similar pattern was observed for the 12-month recall period, with 5% (*OR* = 1.05, 95% CI [0.94, 1.18]) females more likely to report IPV and 18% (*OR* = 1.18, 95% CI [1.04, 1.34]) more likely to report psychological violence in 2022 (Table 2b).

**Table 2a.** Association Between IPV and Explanatory Factors Among Females (Adjusted Odds Ratios – Lifetime).

Variable	%	IPV (All types), lifetime	%	Psychological IPV, lifetime	%	Physical IPV, lifetime	%	Sexual IPV, lifetime
Year								
2014	23.0	Ref	20.7	ref	24.7	Ref	25.1	ref
2022	77.0	1.09 (0.98, 1.21)	79.3	1.31 (1.18, 1.47)***	75.3	0.97 (0.86, 1.08)	74.9	0.95 (0.82, 1.11)
Age								
15–24	15.2	Ref	14.6	ref	14.0	Ref	13.7	ref
25–34	41.0	1.25 (1.10, 1.43)**	40.8	1.27 (1.11, 1.47)**	40.6	1.39 (1.21, 1.59)***	39.7	1.28 (1.04, 1.58)*
35–49	43.8	1.39 (1.20, 1.59)***	44.6	1.44 (1.24, 1.66)***	45.4	1.57 (1.36, 1.80)***	46.6	1.51 (1.23, 1.86)***
Place of residence								
Urban	36.2	Ref	36.4	ref	34.6	Ref	37.1	ref
Rural	63.8	1.12 (1.004, 1.24)*	63.6	1.09 (0.98, 1.21)	65.4	1.11 (0.99, 1.24)	62.9	0.96 (0.82, 1.12)
Education level								
No education	6.4	Ref	5.6	ref	7.5	Ref	5.1	ref
Primary	52.0	1.72 (1.52, 1.96)***	51.7	1.89 (1.64, 2.17)***	55.7	1.38 (1.20, 1.59)***	56.1	1.88 (1.48, 2.39)***
Secondary	30.5	1.47 (1.27, 1.70)***	31.3	1.73 (1.48, 2.03)***	28.9	1.02 (0.87, 1.20)	29.9	1.58 (1.22, 2.06)**
Higher	11.1	0.83 (0.69, 0.997)*	11.5	1.06 (0.86, 1.30)	7.9	0.43 (0.36, 0.53)***	8.9	0.87 (0.60, 1.28)
Perpetrated physical violence against partner								
No	94.0	Ref	93.7	ref	92.6	Ref	90.7	ref
Yes	6.0	13.72 (8.58, 21.94)***	6.3	5.41 (3.90, 7.51)***	7.4	11.09 (7.70, 15.99)***	9.3	4.34 (3.23, 5.83)***
Father beat mother								
No	50.5	Ref	50.4	ref	48.8	Ref	43.6	ref
Yes	43.4	2.24 (2.03, 2.47)***	44.0	1.97 (1.77, 2.19)***	44.9	2.02 (1.84, 2.22)***	50.3	2.13 (1.85, 2.46)***
Do not know	6.1	1.72 (1.39, 2.12)***	5.6	1.37 (1.13, 1.66)**	6.4	1.67 (1.39, 2.02)***	6.1	1.54 (1.09, 2.20)*

*Note.* IPV: intimate partner violence.

Significance ***p < .001. **p < .01. *p < .05.

**Table 2b.** Association Between IPV and Explanatory Factors Among Females (Adjusted Odds Ratios – Past 12 months).

Variable	%	IPV (all types), past 12 months	%	Psychological IPV, past 12 months	%	Physical IPV, past 12 months	%	Sexual IPV, past 12 months
Year								
2014	23.5	ref	21.8	ref	26.5	Ref	27.1	ref
2022	76.5	1.05 (0.94, 1.17)	78.2	1.18 (1.04, 1.33)**	73.5	0.90 (0.79, 1.03)	72.9	0.87 (0.72, 1.04)
Age								
15–24	18.0	ref	16.6	ref	19.3	Ref	17.1	ref
25–34	43.3	1.01 (0.88, 1.16)	42.8	1.11 (0.95, 1.30)	44.4	0.98 (0.85, 1.13)	44.2	1.14 (0.91, 1.42)
35–49	38.7	0.82 (0.70, 0.94)**	40.6	0.996 (0.85, 1.17)	36.4	0.71 (0.61, 0.83)***	38.7	0.95 (0.75, 1.20)
Place of residence								
Urban	35.7	ref	35.4	ref	35.6	Ref	35.8	ref
Rural	64.3	1.14 (1.01, 1.27)*	64.6	1.14 (1.01, 1.29)*	64.4	1.04 (0.91, 1.18)	64.2	1.07 (0.88, 1.29)
Education level								
No education	6.6	ref	5.9	ref	7.5	Ref	5.2	ref
Primary	52.0	1.47 (1.28, 1.69)***	52.6	1.65 (1.41, 1.93)***	56.1	1.29 (1.09, 1.52)**	55.2	1.77 (1.34, 2.34)***
Secondary	31.2	1.30 (1.11, 1.52)**	30.7	1.43 (1.20, 1.71)***	30.3	0.98 (0.82, 1.17)	31.9	1.61 (1.19, 2.18)**
Higher	10.2	0.74 (0.60, 0.91)**	10.9	0.93 (0.73, 1.17)	6.1	0.35 (0.26, 0.45)***	7.7	0.75 (0.45, 1.27)
Perpetrated physical violence against partner								
No	93.7	ref	93.3	ref	91.8	Ref	89.6	ref
Yes	6.3	4.14 (3.10, 5.55)***	6.7	3.75 (2.84, 4.94)***	8.2	4.83 (3.66, 6.37)***	10.4	4.35 (3.13, 6.06)***
Father beat mother								
No	49.8	ref	49.7	ref	49.0	Ref	42.4	ref
Yes	44.2	1.91 (1.72, 2.12)***	44.6	1.81 (1.62, 2.03)***	45.2	1.73 (1.54, 1.93)***	50.8	2.12 (1.81, 2.49)***
Do not know	6.0	1.52 (1.19, 1.94)**	5.7	1.33 (1.08, 1.63)**	5.9	1.33 (1.05, 1.67)*	6.9	1.77 (1.18, 2.67)**

*Note.* IPV: intimate partner violence.

Significance ***p < .001. **p < .01. *p < .05.

Increasing age was significantly associated with higher odds of IPV; women aged 25 to 34 had a 25% increase in the odds of overall IPV (*OR* = 1.25, 95% CI [1.10, 1.43]), which further rose among those aged 35 to 49 (*OR* = 1.39, 95% CI [1.20, 1.59]). Rural residence was also linked to higher IPV risk in females, with rural women having significantly increased odds of lifetime IPV (*OR* = 1.12, 95% CI [1.003, 1.24]) and past year IPV (*OR* = 1.14, 95% CI [1.01, 1.28]). In terms of education, while primary (overall IPV *OR* = 1.72, 95% CI [1.51, 1.96]) and secondary education (overall IPV *OR* = 1.47, 95% CI [1.26, 1.70]) were associated with elevated IPV risk compared to no education, higher education was significantly associated with reduced odds of lifetime physical IPV (*OR* = 0.43, 95% CI [0.36, 0.53]) and with lower odds of any IPV in the past year (*OR* = 0.74, 95% CI [0.60, 0.91]) and past year physical IPV (*OR* = 0.35, 95% CI [0.26, 0.45]). With the exception of past year IPV in 2022, there were a few differences in the associations for specific variables in the year-specific analyses (Supplemental Tables S1–S4).

### Males

The prevalence of IPV was slightly higher in 2022 for all categories (Table 1). A higher prevalence was reported for any form of lifetime IPV in 2022 (23.4% vs. 29.7%); and psychological IPV (20.4% vs. 26.5%). During the 12-month recall period, 17.4% and 19.3% of men reported experiencing any form of IPV in 2014 and 2022, respectively. Psychological violence was the most frequently reported type of IPV during the lifetime and 12 months before the survey. The proportion of men who initiated physical violence against their partner was lower in 2022 (37.6% vs. 25.4%). Similarly, men who reported that their fathers had beat their mothers were 48.8% in 2014 versus 42.0% in 2022.

**Figure 2.**
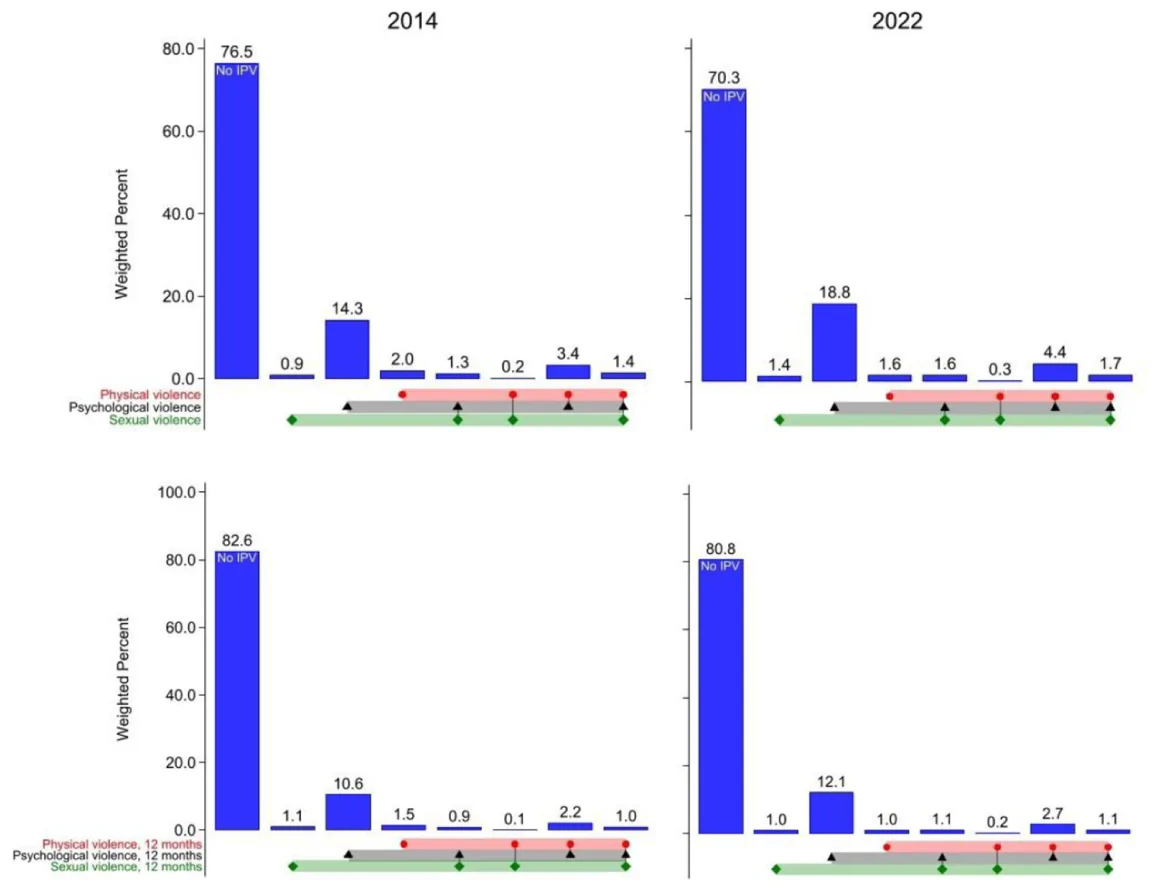
Profile of IPV types for males – Lifetime (above) and past 12 months (below). *Note.* Vertical bars represent the prevalence of respective combinations of types of IPV with non-missing numbers. IPV: intimate partner violence.

Concerning multiple forms of violence, 1.4% and 1.7% of males reported experiencing all three types of violence during their lifetime in 2014 and 2022, respectively (Figure 2). The most frequent combination of IPV type was physical and psychological violence at 3.4% in 2014 and 4.4% in 2022, while the least frequent type was physical with sexual violence both in 2014 at 0.2% and 0.3% in 2022. During the 12-month recall period, the men who reported three forms of violence were 1.0% in 2014 and 1.1% in 2022. Congruently, a combination of physical and psychological violence was the most frequent during both time periods at 2.2% and 2.7% in 2014 and 2022, respectively, while the least frequent was physical with sexual violence.

The odds of reported lifetime IPV for men was higher in 2022 (Table 3a). The lifetime odds were 78% higher for any IPV (*OR* = 1.78, 95% CI [1.50, 2.12]), 77% higher for psychological IPV (*OR* = 1.77, 95% CI [1.48, 2.12]), 47% higher for physical IPV (*OR* = 1.47, 95% CI [1.12, 1.94]), and 69% higher for sexual violence (*OR* = 1.68, 95% CI [1.16, 2.46]) in 2022. During the past year of 2022, men were 38% more likely to report any form of IPV (*OR* = 1.38, 95% CI [1.14, 1.66]), and 43% more likely to report psychological IPV (*OR* = 1.43, 95% CI [1.18, 1.73]) (Table 3b). Although not statistically significant, the odds of reporting physical and sexual violence were also higher in 2022.

**Table 3a.** Association Between IPV and Explanatory Factors Among Males (Adjusted Odds Ratios – Lifetime).

Variable	%	IPV (All types), lifetime	%	Psychological IPV, lifetime	%	Physical IPV, lifetime	%	Sexual IPV, lifetime
Year								
2014	40.9	ref	40.2	ref	43.9	Ref	40.2	ref
2022	59.1	1.78 (1.50, 2.12)***	59.8	1.77 (1.48, 2.12)***	56.1	1.47 (1.12, 1.94)**	59.8	1.68 (1.16, 2.45)**
Age								
15–24	8.0	ref	7.4	ref	9.6	Ref	15.4	ref
25–34	38.8	0.79 (0.56, 1.11)	39.4	0.92 (0.64, 1.33)	34.2	0.62 (0.34, 1.14)	43.9	0.49 (0.26, 0.90)*
35–44	33.7	0.71 (0.51, 0.98)*	33.8	0.81 (0.57, 1.17)	33.5	0.63 (0.35, 1.14)	27.4	0.31 (0.16, 0.59)***
45–54	19.5	0.54 (0.38, 0.77)**	19.4	0.62 (0.42, 0.92)*	22.7	0.58 (0.31, 1.10)	13.4	0.21 (0.10, 0.45)***
Place of residence								
Urban	42.1	ref	41.6	ref	41.4	Ref	48.4	ref
Rural	57.9	0.94 (0.78, 1.13)	58.4	0.98 (0.80, 1.19)	58.6	0.92 (0.70, 1.21)	51.6	0.73 (0.52, 1.04)
Education level								
No education	2.5	ref	2.5	ref	2.0	Ref	1.9	ref
Primary	49.5	1.84 (1.25, 2.71)**	49.5	1.76 (1.16, 2.65)**	51.5	2.12 (1.11, 4.03)*	49.8	2.12 (1.02, 4.40)*
Secondary	31.1	1.93 (1.29, 2.90)**	30.2	1.77 (1.16, 2.70)**	33.3	2.45 (1.27, 4.74)**	33.4	2.18 (0.99, 4.78)
Higher	16.9	1.74 (1.13, 2.68)*	17.8	1.79 (1.13, 2.83)*	13.2	1.74 (0.83, 3.61)	15.0	1.62 (0.71, 3.73)
Perpetrated physical violence against partner								
No	47.3	ref	47.7	ref	34.6	Ref	40.7	ref
Yes	52.7	3.98 (3.36, 4.72)***	52.3	3.67 (3.07, 4.40)***	65.4	4.43 (3.30, 5.95)***	59.3	3.41 (2.37, 4.90)***
Father beat mother								
No	42.6	ref	43.4	ref	30.3	Ref	37.1	ref
Yes	51.8	1.23 (1.03, 1.47)*	51.0	1.16 (0.97, 1.39)	64.8	1.94 (1.45, 2.59)***	59.8	1.58 (1.08, 2.32)*
Do not know	5.5	0.94 (0.69, 1.30)	5.6	0.95 (0.68, 1.32)	4.9	1.14 (0.60, 2.14)	3.1	0.61 (0.26, 1.39)

*Note.* IPV: intimate partner violence.

Significance ***p < 0.001, **p < .01, *p < .05.

**Table 3b.** Association Between IPV and Explanatory Factors Among Males (Adjusted Odds Ratios – 12 months).

Variable	%	IPV (All types), past 12 months	%	Psychological IPV, past 12 months	%	Physical IPV, past 12 months	%	Sexual IPV, past 12 months
Year								
2014	44.2	ref	43.1	Ref	45.6	Ref	44.4	ref
2022	55.8	1.38 (1.14, 1.66)**	56.9	1.43 (1.18, 1.73)*	54.4	1.35 (0.97, 1.88)	55.6	1.40 (0.92, 2.14)
Age								
15–24	10.0	ref	9.5	Ref	11.3	Ref	16.6	ref
25–34	41.3	0.63 (0.44, 0.91)*	40.8	0.68 (0.46, 1.02)	38.1	0.60 (0.30, 1.20)	51.9	0.55 (0.27, 1.11)
35–44	31.6	0.49 (0.34, 0.70)***	32.3	0.56 (0.37, 0.84)**	30.4	0.49 (0.24, 0.98)*	23.4	0.26 (0.12, 0.56)**
45–54	17.1	0.35 (0.24, 0.52)***	17.3	0.40 (0.26, 0.61)***	20.2	0.44 (0.21, 0.94)*	8.0	0.12 (0.05, 0.28)***
Place of residence								
Urban	42.4	ref	41.3	Ref	44.6	Ref	54.5	ref
Rural	57.6	0.97 (0.79, 1.17)	58.7	1.02 (0.82, 1.26)	55.4	0.83 (0.60, 1.14)	45.5	0.62 (0.42, 0.92)*
Education leve**l**								
No education	2.2	ref	2.4	Ref	2.0	Ref	1.3	ref
Primary	49.5	1.94 (1.22, 3.07)**	49.8	1.76 (1.09, 2.86)*	49.8	1.94 (0.86, 4.35)	45.8	2.77 (1.004, 7.63)*
Secondary	30.9	2.01 (1.23, 3.29)**	29.8	1.75 (1.05, 2.90)*	34.8	2.32 (1.02, 5.31)*	36.3	3.26 (1.13, 9.39)*
Higher	17.4	1.95 (1.16, 3.27)*	18.0	1.85 (1.07, 3.18)*	13.4	1.62 (0.64, 4.07)	16.7	2.50 (0.83, 7.52)
Perpetrated physical violence against partner								
No	47.2	ref	47.7	Ref	35.4	Ref	39.9	ref
Yes	52.8	3.21 (2.66, 3.89)***	52.3	3.05 (2.48, 3.74)***	64.6	4.03 (2.80, 5.80)***	60.1	3.44 (2.26, 5.24)***
Father beat mother								
No	41.2	ref	41.9	Ref	29.5	Ref	36.3	ref
Yes	53.8	1.34 (1.09, 1.64)**	53.2	1.27 (1.03, 1.56)*	64.6	2.02 (1.42, 2.88)***	60.0	1.74 (1.09, 2.78)*
Do not know	5.0	0.88 (0.60, 1.30)	4.9	0.85 (0.57, 1.29)	5.9	1.47 (0.70, 3.06)	3.7	0.79 (0.32, 1.94)

*Note.* IPV: intimate partner violence.

Significance ***p < .001. **p < 0.01. *p < .05.

The older age groups had significantly lower odds of IPV compared with those aged 15 to 24; specifically, males aged 35 to 44 experienced a 30% reduction in the odds of overall IPV (*OR* = 0.70, 95% CI [0.51, 0.98]) and a notable reduction in sexual violence (*OR* = 0.31, 95% CI [0.16, 0.59]), while those aged 45 to 54 had even lower odds of overall IPV (*OR* = 0.54, 95% CI [0.38, 0.77]), sexual violence (*OR* = 0.21, 95% CI [0.10, 0.45]), and any IPV in the past year (*OR* = 0.35, 95% CI [0.24, 0.52]). In contrast, higher educational attainment among males was associated with significantly increased odds of IPV; for example, compared with men with no education, those with primary education had higher odds of overall IPV (*OR* = 1.84, 95% CI [1.25, 2.71]) and sexual violence (*OR* = 2.12, 95% CI [1.02, 4.40]), and similar elevated associations were observed for secondary (overall IPV *OR* = 1.93, 95% CI [1.29, 2.90]) and higher education (overall IPV *OR* = 1.74, 95% CI [1.13, 2.68]). Men living in rural areas were 38% less likely to report sexual IPV in the past 12 months (*OR* = 0.62, 95% CI [0.42, 0.92]). A few differences were observed in the associations for specific variables in the year-specific analyses (Supplemental Tables S5–S8).

## Discussion

This article presents estimates of self-reported IPV among females and males in Kenya using nationally representative data collected pre- and during COVID-19. The key findings are: first, among females, the overall lifetime and recent reports of IPV remained largely unchanged, although there was a notable shift in the type of violence, with psychological IPV increasing while physical IPV declined. Second, males reported an increase in IPV across both lifetime and 12-month recall periods, with psychological violence emerging as the most common form. Third, sociodemographic factors associated with IPV included age, education level, and place of residence, parental violence and initiating physical violence.

The finding of no change in any form of IPV against females in 2014 and 2022 for the lifetime and 12-month recall periods could be due to changes in the prevalence of specific forms of IPV. For instance, a decline in physical and sexual violence and an increase in psychological IPV could balance off, leading to a no-difference observation overall. The decline in physical and sexual IPV against females in 2022 could be attributed to the various mitigation measures that have been put in place (Arango et al., 2014; Ellsberg et al., 2015; Torres-Rueda et al., 2020). Physical IPV is well recognized by both victims and perpetrators; it is the form of violence that causes obvious bodily harm, whose effects are overt, making it the form that often attracts punishment. Being a male-dominated society, women have often been viewed as being subordinate to men (Bah & Barasa, 2023; Miruka et al., 2024), a notion that has been challenged by social and economic changes, which have been highly influenced and driven by policy and legal frameworks that advocate for gender equality.

It is also possible that the reported decline in the physical and sexual IPV prevalence may be a result of social desirability bias rather than real decline because of greater awareness of IPV through GBV programs and campaigns. With improved legal and justice systems, men are more likely to be punished for perpetrating violence against their female partners (Ikuteyijo et al., 2024; Judiciary, 2023). The increase in lifetime and 12-month recall period psychological violence against women might be attributable to the adoption of less violent and overt forms of aggression by men, which are less likely to attract legal action. This might explain psychological IPV as the most prevalent form of violence. This also raises uncertainties if fundamental changes in power relations, mindsets, and behavior occurred or if superficial changes shifted from overt physical IPV to subtle, less visible psychological forms of IPV without transforming the underlying power dynamics and attitudes.

The IPV estimates for male victimization showed an increase in 2022. During the pandemic, masculinity and gender roles may have been challenged with some men losing jobs and income, especially those in daily low-pay employment (Jewkes et al., 2015; Reed et al., 2023). Rigid gender roles expect men to be “breadwinners” and not being able to live up to such expectations can trigger conflict and violence within relationships, potentially increasing female perpetrated violence (Reed et al., 2023). It was also possible that the rise violence reported by men was reactive arising from unresolved conflicts, economic stress, or substance use (Waila et al., 2024), which our study did not investigate. In the Kenyan setting, gender-based violence is often interpreted as focusing on women because men are deemed to be strong both physically and emotionally, hence unlikely to experience female-perpetrated violence. Therefore, violence prevention interventions have focused on female victims, an approach that leaves men vulnerable to female abuse (Bates, 2016; Tenkorang et al., 2023; Waila et al., 2024). Extant literature shows that although social and economic empowerment of women shields them from IPV (Bandiera et al., 2018; Bhalotra et al., 2021; Eggers Del Campo & Steinert, 2022), the shift in power dynamics in a society that seem stuck on patriarchy can precipitate violence against men once women start to question long-standing and culturally backed gender roles and norms (Waila et al., 2024).

We acknowledge that our IPV prevalence estimates against males might still be an underestimation of the true magnitude of the problem since men have been shown to underreport their exposure to female-perpetrated IPV and reluctance to seek help (Tenkorang et al., 2023). However, we cannot rule out the possibility that men may have also become more willing to disclose their IPV. This could be linked to an increase in the severity or frequency of IPV victimization over time, or because they have become more aware that they are potential victims of female-perpetrated IPV. Furthermore, owing to their muscular weakness, except when they use objects or weapons to inflict physical harm, women are more psychologically abusive toward their male intimate partners, a form of violence that has devastating effects (Lagdon et al., 2014; Waila et al., 2024).

Additionally, an increase in IPV during the COVID-19 pandemic observed in other studies (Ghahramani et al., 2024; Kifle et al., 2024) including in Kenya (Ahmed et al., 2021) was not observed in this study among women. Additionally higher rates of IPV were reported in Kenya at the beginning of the pandemic could be attributed to reduced access to support services, economic stressors, and curfews (Ahmed et al., 2021; Reed et al., 2023). The already high pre-COVID IPV prevalence rates among women in Kenya and cultural attitudes could have contributed to the unobserved change or effect.

The observed associations between sociodemographic factors and IPV in Kenya can be understood through a framework that integrates cultural, economic, and power dynamics. In the Kenyan context, traditional patriarchal norms and gender inequalities persist, influencing both the perpetration and tolerance of IPV. For instance, women with lower levels of education may lack the economic and social empowerment necessary to challenge or escape abusive relationships, whereas higher educational attainment appears to provide women with greater agency and awareness of their rights (Ikwara et al., 2025; Melkam et al., 2024). Conversely, among men, higher education might be linked with different expressions of masculinity or increased reporting of IPV, while younger age may correspond with more impulsive or aggressive behaviors that diminish over time with maturity and changing societal attitudes. Additionally, rural residence is often associated with limited access to formal support services and entrenched cultural norms that may tacitly condone or normalize IPV, further exacerbating women’s vulnerability (Hatcher et al., 2013; Mkutu et al., 2020). Additionally, research has challenged the dichotomy of rural versus urban IPV rates (Nabaggala et al., 2021). Treating “urban” as a homogeneous space, as the DHS does, overlooks the fact that urban locations encompass both affluent and deprived areas (Atkinson, 2024; Brown et al., 2018). People in informal urban settlements were potentially underrepresented in the DHS but potentially hit hardest during crises such as the COVID-19 pandemic and these restrictions that could trigger IPV (Brown et al., 2018). Overall, these findings are consistent with previous studies in Kenya and sub-Saharan Africa (Hatcher et al., 2013; Ikwara et al., 2025; Melkam et al., 2024; Mkutu et al., 2020), which highlight the interplay between sociodemographic factors and cultural norms in shaping IPV dynamics.

### Limitations and Strengths

Limitations: First, this study relies on cross-sectional survey data from 2014 and 2022, which limits causal inferences. Second, IPV is a highly sensitive subject, and underreporting may have occurred and been likely due to recall bias and social desirability, particularly among men. Gender based violence (GBV) prevention programs may have contributed to social desirability bias and/or a decline in reports of IPV. Third, our findings did not specifically estimate IPV among vulnerable populations, such as those in informal settlements or those with disability; thus, in comparison to our estimates, the true estimates among special or diverse groups may be under or overrepresented. Fourth, the Kenyan 7th DHS standard questionnaire was not designed to capture COVID‑specific triggers related to IPV in general and within specific vulnerable populations, for example in informal settlements, which represents a missed opportunity to directly estimate pandemic‑related influences on IPV.

Strengths: The study draws on two waves of nationally representative data, ensuring high-quality and reliable estimates of IPV in Kenya. Due to the sample that is nationally representative, the overall findings can be generalized to the entire country, including the East African region. It also includes sex-disaggregated analyses across psychological, physical, and sexual domains, thus contributing knowledge of how violence affects both sexes. The use of the same standardized IPV measurement tools across survey rounds enhances comparability over time. Together, these strengths support the validity of the findings and underscore the importance of expanding gender-inclusive IPV research and prevention programming in Kenya and across similar settings in the East African region.

## Conclusion

Except for psychological IPV, our study shows no significant differences for any other form of IPV against women in 2022 relative to 2014. However, female-to-male IPV seems to be on the rise, with more men reporting exposure to any form of IPV. It is possible that IPV prevention interventions in Kenya have concentrated on women as victims, leaving out men who are also subjected to female-perpetrated IPV. Cognizant of the gains made in the fight against violence against women, we posit that gender-sensitive IPV prevention strategies, which recognize men as potential victims of IPV, would be more responsive in a highly dynamic social and economic environment.

## Supplemental Material

sj-docx-1-jiv-10.1177_08862605261444013 – Supplemental material for Sex-Specific Changes and Correlates of Intimate Partner Violence in Kenya: Analyses of Nationally Representative Surveys From 2014 and 2022 Supplemental material, sj-docx-1-jiv-10.1177_08862605261444013 for Sex-Specific Changes and Correlates of Intimate Partner Violence in Kenya: Analyses of Nationally Representative Surveys From 2014 and 2022 by Anne Abio, Mahsa MohammadNamdar, Omid Dadras, Michael Lowery Wilson and Jacinta Mukulu Waila in Journal of Interpersonal Violence
