# Supplementary material for: Sex-Specific Changes and Correlates of Intimate Partner Violence in Kenya: Analyses of Nationally Representative Surveys From 2014 and 2022
Source: J Interpers Violence. 2026 May 30;41(19-20):7923–48. doi: 10.1177/08862605261444013 (PMC13570972; doi:10.1177/08862605261444013)
Supplement: Supplementary file 1 [file sj-docx-1-jiv-10.1177_08862605261444013.docx]

**Sex-specific changes and correlates of intimate partner violence in Kenya: analyses of nationally representative surveys from 2014 and 2022**

**Supplementary tables**

**Table S1: Association between IPV and explanatory factors among females (Adjusted Odds Ratios – Lifetime in 2014)**

|  | **%** | **IPV (All types), lifetime** | **%** | **Psychological IPV, lifetime** | **%** | **Physical IPV, lifetime** | **%** | **Sexual IPV, lifetime** |
| --- | --- | --- | --- | --- | --- | --- | --- | --- |
| **Age** |  |  |  |  |  |  |  |  |
| 15-24 | 16.0 | ref | 15.3 | ref | 15.9 | ref | 16.2 | ref |
| 25-34 | 45.0 | 1.49 (1.17, 1.88)** | 44.4 | 1.42 (1.09, 1.85)* | 43.9 | 1.39 (1.08, 1.79)* | 44.5 | 1.28 (0.86, 1.91)‡ |
| 35-49 | 39.1 | 1.50 (1.17, 1.93)** | 40.3 | 1.57 (1.20, 2.04)** | 40.2 | 1.49 (1.13, 1.96)** | 39.3 | 1.33 (0.89, 2.00)‡ |
| **Place of residence** | | |  |  |  |  |  |  |
| Urban | 38.8 | ref | 39.4 | ref | 35.9 | ref | 41.8 | ref |
| Rural | 61.2 | 0.95 (0.78, 1.15)‡ | 60.6 | 0.95 (0.78, 1.17) | 64.1 | 1.10 (0.88, 1.36) | 58.2 | 0.77 (0.58, 1.01) |
| **Education level** | | |  |  |  |  |  |  |
| No education | 7.4 | ref | 6.5 | ref | 8.3 | ref | 6.5 | ref |
| Primary | 61.5 | 1.70 (1.34, 2.16)*** | 61.3 | 1.83 (1.40, 2.40)*** | 64.1 | 1.45 (1.11, 1.90)** | 67.3 | 1.72 (1.14, 2.59)* |
| Secondary | 25.1 | 1.45 (1.09, 1.93)* | 25.4 | 1.65 (1.20, 2.28)** | 23.3 | 1.09 (0.79, 1.51) | 22.0 | 1.19 (0.73, 1.93)‡ |
| Higher | 6.0 | 0.84 (0.56, 1.29)‡ | 6.8 | 1.24 (0.76, 2.01) | 4.2 | 0.47 (0.28, 0.80)** | 4.3 | 0.65 (0.30, 1.41) |
| **Perpetrated physical violence against partner** | | |  | |  | | |  |
| No | 92.6 | ref | 91.5 | ref | 91.0 | ref | 88.5 | ref |
| Yes | 7.4 | 13.26 (5.8, 30.12)*** | 8.5 | 6.02 (3.90, 9.30)*** | 9.0 | 14.36 (7.18, 28.73)*** | 11.5 | 4.67 (2.82, 7.74)*** |
| **Father beat mother** | | |  |  |  |  |  |  |
| No | 48.2 | ref | 49.1 | ref | 46.6 | ref | 40.9 | ref |
| Yes | 45.4 | 1.87 (1.57, 2.23)*** | 45.3 | 1.55 (1.29, 1.86)*** | 46.9 | 1.78 (1.49, 2.13)*** | 53.3 | 2.04 (1.56, 2.66)*** |
| Don't know | 6.4 | 1.40 (1.001, 1.97)‡ | 5.6 | 1.03 (0.69, 1.53)‡ | 6.5 | 1.41 (0.998, 1.99)‡ | 5.8 | 1.27 (0.76, 2.13)‡ |

Significance ***p<0.001, **p<0.01, *p<0.05

Change in association compared to Table 2a ‡

**Table S2: Association between IPV and explanatory factors among females (Adjusted Odds Ratios – Lifetime in 2022)**

|  | **%** | **IPV (All types), lifetime** | **%** | **Psychological IPV, lifetime** | **%** | **Physical IPV, lifetime** | **%** | **Sexual IPV, lifetime** |
| --- | --- | --- | --- | --- | --- | --- | --- | --- |
| **Age** |  |  |  |  |  |  |  |  |
| 15-24 | 15.0 | ref | 14.4 | ref | 13.4 | ref | 12.9 | ref |
| 25-34 | 39.9 | 1.19 (1.01, 1.39)* | 39.9 | 1.23 (1.05, 1.45)* | 39.5 | 1.39 (1.18, 1.63)*** | 38.1 | 1.30 (1.02, 1.65)* |
| 35-49 | 45.2 | 1.35 (1.14, 1.60)*** | 45.7 | 1.41 (1.19, 1.67)*** | 47.1 | 1.59 (1.35, 1.88)*** | 49.0 | 1.60 (1.26, 2.04)*** |
| **Place of residence** | | |  |  |  |  |  |  |
| Urban | 35.4 | ref | 35.7 | ref | 34.2 | ref | 35.5 | ref |
| Rural | 64.6 | 1.17 (1.04, 1.33)* | 64.3 | 1.13 (1.001, 1.27)*‡ | 65.8 | 1.11 (0.97, 1.27) | 64.5 | 1.04 (0.85, 1.26) |
| **Education level** | | |  |  |  |  |  |  |
| No education | 6.1 | ref | 5.3 | ref | 7.2 | ref | 4.7 | ref |
| Primary | 49.2 | 1.72 (1.48, 2.00)*** | 49.2 | 1.91 (1.62, 2.24)*** | 52.9 | 1.36 (1.15, 1.60)*** | 52.3 | 1.93 (1.44, 2.59)*** |
| Secondary | 32.2 | 1.46 (1.23, 1.73)* | 32.8 | 1.75 (1.46, 2.10)*** | 30.8 | 1.0002 (0.83, 1.20) | 32.6 | 1.74 (1.27, 2.38)** |
| Higher | 12.6 | 0.83 (0.67, 1.02)‡ | 12.7 | 1.04 (0.83, 1.31) | 9.2 | 0.43 (0.34, 0.53)*** | 10.5 | 0.95 (0.62, 1.47) |
| **Perpetrated physical violence against partner** | | |  | |  | | |  |
| No | 94.4 | ref | 94.3 | ref | 93.1 | ref | 91.5 | ref |
| Yes | 5.6 | 13.68 (7.72, 24.24)*** | 5.8 | 5.08 (3.34, 7.73)*** | 6.9 | 10.11 (6.56, 15.57)*** | 8.5 | 4.12 (2.87, 5.93)*** |
| **Father beat mother** | | |  |  |  |  |  |  |
| No | 51.2 | ref | 50.8 | ref | 49.4 | ref | 44.5 | ref |
| Yes | 42.8 | 2.37 (2.11, 2.66)*** | 43.6 | 2.11 (1.87, 2.39)*** | 44.3 | 2.10 (1.88, 2.34)*** | 49.3 | 2.17 (1.83, 2.57)*** |
| Don't know | 6.0 | 1.84 (1.42, 2.39)*** | 5.6 | 1.50 (1.21, 1.87)*** | 6.3 | 1.78 (1.42, 2.23)*** | 6.1 | 1.65 (1.07, 2.55)* |

Significance ***p<0.001, **p<0.01, *p<0.05

Change in association compared to Table 2a ‡

**Table S3: Association between IPV and explanatory factors among females (Adjusted Odds Ratios – Past 12 months in 2014)**

|  | **%** | **IPV (All types), past 12 months** | | **%** | | **Psychological IPV, past 12 months** | | **%** | | **Physical IPV, past 12 months** | | **%** | | **Sexual IPV, past 12 months** | |  |
| --- | --- | --- | --- | --- | --- | --- | --- | --- | --- | --- | --- | --- | --- | --- | --- | --- |
| **Age** |  |  | |  | |  | |  | |  | |  | |  | |  |
| 15-24 | 18.5 | ref | | 17.8 | | ref | | 19.4 | | ref | | 18.4 | | ref | |  |
| 25-34 | 46.4 | 1.16 (0.90, 1.49) | | 45.1 | | 1.13 (0.86, 1.50)‡ | | 46.5 | | 1.09 (0.83, 1.43) | | 46.2 | | 1.15 (0.74, 1.78) | |  |
| 35-49 | 35.2 | 0.96 (0.73, 1.27)‡ | | 37.1 | | 1.10 (0.82, 1.47)‡ | | 34.2 | | 0.86 (0.63, 1.18)‡ | | 35.5 | | 1.02 (0.63, 1.64) | |  |
| **Place of residence** | | |  | |  | |  | |  | |  | |  | |  | |
| Urban | 41.0 | ref | | 41.6 | | ref | | 38.1 | | ref | | 44.3 | | ref | |  |
| Rural | 59.0 | 0.87 (0.71, 1.07)‡ | | 58.4 | | 0.87 (0.70, 1.10)‡ | | 61.9 | | 0.99 (0.77, 1.26) | | 55.8 | | 0.68 (0.49, 0.95)*‡ | |  |
| **Education level** | | |  | |  | |  | |  | |  | |  | |  | |
| No education | 7.5 | ref | | 6.8 | | ref | | 8.9 | | ref | | 6.9 | | ref | |  |
| Primary | 60.6 | 1.44 (1.11, 1.87)** | | 60.7 | | 1.59 (1.18, 2.15)** | | 63.6 | | 1.18 (0.86, 1.61)‡ | | 68.5 | | 1.57 (0.98, 2.53)‡ | |  |
| Secondary | 25.8 | 1.31 (0.96, 1.78)‡ | | 25.4 | | 1.43 (1.01, 2.02)* | | 23.8 | | 0.95 (0.65, 1.38) | | 21.0 | | 1.01 (.58, 1.75)‡ | |  |
| Higher | 6.0 | 0.80 (0.48, 1.32)‡ | | 7.2 | | 1.19 (0.69, 2.03) | | 3.7 | | 0.37 (0.17, 0.79)* | | 3.8 | | 0.51 (0.20, 1.32) | |  |
| **Perpetrated physical violence against partner** | | | | |  | |  | |  | |  | |  | | | |
| No | 91.4 | ref | | 90.6 | | ref | | 89.2 | | ref | | 87.8 | | ref | |  |
| Yes | 8.6 | 6.01 (3.78, 9.55)*** | | 9.4 | | 4.74 (3.15, 7.13)*** | | 10.8 | | 6.66 (3.83, 11.59)*** | | 12.2 | | 4.30 (2.58, 7.16)*** | |  |
| **Father beat mother** | | | | |  | |  | |  | |  | |  | |  | |
| No | 48.9 | ref | | 49.8 | | ref | | 47.2 | | ref | | 38.5 | | ref | |  |
| Yes | 45.2 | 1.60 (1.33, 1.91)*** | | 45.2 | | 1.46 (1.20, 1.78)*** | | 46.4 | | 1.56 (1.27, 1.92)*** | | 55.9 | | 2.25 (1.66, 3.05)*** | |  |
| Don't know | 5.9 | 1.15 (0.78, 1.69)‡ | | 5.0 | | 0.89 (0.56, 1.42)‡ | | 6.4 | | 1.26 (0.80, 1.97)‡ | | 5.6 | | 1.29 (0.72, 2.31)‡ | |  |

Significance ***p<0.001, **p<0.01, *p<0.05

Change in association compared to Table 2b ‡

**Table S4: Association between IPV and explanatory factors among females (Adjusted Odds Ratios – Past 12 months in 2022)**

|  | **%** | **IPV (All types), past 12 months** | | **%** | | **Psychological IPV, past 12 months** | | **%** | | **Physical IPV, past 12 months** | | **%** | | **Sexual IPV, past 12 months** | |  |
| --- | --- | --- | --- | --- | --- | --- | --- | --- | --- | --- | --- | --- | --- | --- | --- | --- |
| **Age** |  |  | |  | |  | |  | |  | |  | |  | |  |
| 15-24 | 17.9 | ref | | 16.2 | | ref | | 19.2 | | ref | | 16.6 | | ref | |  |
| 25-34 | 42.4 | 0.96 (0.81, 1.13) | | 42.2 | | 1.10 (0.92, 1.33) | | 43.6 | | 0.93 (0.79, 1.11) | | 43.5 | | 1.15 (0.89, 1.49) | |  |
| 35-49 | 39.7 | 0.78 (0.65, 0.92)** | | 41.6 | | 0.97 (0.80, 1.18) | | 37.2 | | 0.67 (0.56, 0.79)*** | | 39.9 | | 0.96 (0.73, 1.25) | |  |
| **Place of residence** | | |  | |  | |  | |  | |  | |  | |  | |
| Urban | 34.1 | ref | | 33.7 | | ref | | 34.7 | | ref | | 32.6 | | ref | |  |
| Rural | 65.9 | 1.23 (1.08, 1.41)** | | 66.3 | | 1.24 (1.08, 1.42)** | | 65.3 | | 1.05 (0.90, 1.23) | | 67.4 | | 1.26 (0.99, 1.60) | |  |
| **Education level** | | |  | |  | |  | |  | |  | |  | |  | |
| No education | 6.3 | ref | | 5.7 | | ref | | 7.0 | | ref | | 4.6 | | ref | |  |
| Primary | 49.3 | 1.48 (1.25, 1.74)*** | | 50.3 | | 1.67 (1.38, 2.00)*** | | 53.4 | | 1.34 (1.10, 1.62)** | | 50.3 | | 1.85 (1.31, 2.61)** | |  |
| Secondary | 32.9 | 1.28 (1.07, 1.53)** | | 32.2 | | 1.42 (1.16, 1.74)** | | 32.4 | | 0.99 (0.80, 1.22) | | 36.0 | | 1.86 (1.30, 2.66)** | |  |
| Higher | 11.5 | 0.74 (0.59, 0.92)** | | 11.9 | | 0.90 (0.70, 1.17) | | 7.0 | | 0.35 (0.26, 0.46)*** | | 9.1 | | 0.86 (0.47, 1.56) | |  |
| **Perpetrated physical violence against partner** | | | | |  | |  | |  | |  | |  | | | |
| No | 94.4 | ref | | 94.0 | | ref | | 92.7 | | ref | | 90.3 | | ref | |  |
| Yes | 5.6 | 3.56 (2.49, 5.10)*** | | 6.0 | | 3.36 (2.39, 4.73)*** | | 7.3 | | 4.23 (3.03, 5.91)*** | | 9.7 | | 4.26 (2.79, 6.52)*** | |  |
| **Father beat mother** | | | | |  | |  | |  | |  | |  | |  | |
| No | 50.1 | ref | | 49.7 | | ref | | 49.6 | | ref | | 43.8 | | ref | |  |
| Yes | 43.9 | 2.02 (1.79, 2.30)*** | | 44.4 | | 1.93 (1.69, 2.21)*** | | 44.7 | | 1.79 (1.56, 2.05)*** | | 48.9 | | 2.07 (1.71, 2.50)*** | |  |
| Don't know | 6.1 | 1.69 (1.25, 2.27)** | | 5.8 | | 1.51 (1.19, 1.92)** | | 5.7 | | 1.37 (1.04, 1.79)* | | 7.4 | | 1.98 (1.21, 3.26)** | |  |

Significance ***p<0.001, **p<0.01, *p<0.05

Change in association compared to Table 2b ‡

**Table S5: Association between intimate partner violence and explanatory factors among males (Adjusted Odds Ratios – Lifetime in 2014)**

|  | **%** | **IPV (All types), lifetime** | **%** | **Psychological IPV, lifetime** | **%** | **Physical IPV, lifetime** | **%** | **Sexual IPV, lifetime** |
| --- | --- | --- | --- | --- | --- | --- | --- | --- |
| **Age** |  |  |  |  |  |  |  |  |
| 15-24 | 6.7 | ref | 5.6 | ref | 10.1 | ref | 7.4 | ref |
| 25-34 | 44.9 | 1.06 (0.61, 1.87) | 45.2 | 1.32 (0.69, 2.50) | 38.5 | 0.56 (0.27, 1.18) | 53.1 | 1.15 (0.52, 2.56)‡ |
| 35-44 | 31.4 | 0.81 (0.46, 1.42)‡ | 31.6 | 1.02 (0.53, 1.95) | 29.8 | 0.50 (0.24, 1.06) | 26.9 | 0.67 (0.28, 1.59)‡ |
| 45-54 | 17.1 | 0.54 (0.30, 0.992)* | 17.5 | 0.72 (0.37, 1.41)‡ | 21.7 | 0.47 (0.22, 1.03) | 12.5 | 0.43 (0.19, 0.98)* |
| **Place of residence** | | |  |  |  |  |  |  |
| Urban | 42.8 | ref | 42.2 | ref | 42.5 | ref | 48.6 | ref |
| Rural | 57.2 | 1.16 (0.89, 1.49) | 57.9 | 1.20 (0.90, 1.59) | 57.5 | 1.08 (0.74, 1.57) | 51.4 | 0.79 (0.47, 1.33) |
| **Education level** | | |  |  |  |  |  |  |
| No education | 2.1 | ref | 2.2 | ref | 1.5 | ref | 1.3 | ref |
| Primary | 52.4 | 2.11 (1.17, 3.78)* | 52.1 | 1.91 (1.03, 3.54)* | 52.0 | 2.49 (0.85, 7.32)‡ | 58.4 | 3.12 (0.84, 11.54)‡ |
| Secondary | 29.9 | 2.36 (1.27, 4.38)** | 28.8 | 2.05 (1.07, 3.92)* | 33.3 | 3.12 (1.03, 9.51)* | 27.1 | 2.61 (0.67, 10.20) |
| Higher | 15.7 | 3.07 (1.58, 5.90)** | 16.9 | 3.05 (1.53, 6.06)** | 13.2 | 3.08 (0.94, 10.07) | 13.3 | 2.85 (0.69, 11.67) |
| **Perpetrated physical violence against partner** | | | | |  |  |  |  |
| No | 38.4 | ref | 38.0 | ref | 30.7 | ref | 40.5 | ref |
| Yes | 61.6 | 3.85 (3.03, 4.90)*** | 62.0 | 3.77 (2.91, 4.88)*** | 69.3 | 3.94 (2.62, 5.91)*** | 59.6 | 2.44 (1.49, 3.99)*** |
| **Father beat mother** | | |  |  |  |  |  |  |
| No | 38.6 | ref | 39.9 | ref | 30.7 | ref | 29.2 | ref |
| Yes | 56.4 | 1.30 (1.01, 1.68)* | 54.8 | 1.17 (0.90, 1.52) | 64.2 | 1.71 (1.12, 2.61)* | 65.7 | 2.04 (1.16, 3.61)* |
| Don't know | 5.1 | 0.84 (0.52, 1.36) | 5.2 | 0.86 (0.52, 1.42) | 5.1 | 1.10 (0.48, 2.56) | 5.1 | 1.21 (0.38, 3.81) |

Significance ***p<0.001, **p<0.01, *p<0.05

Change in association compared to Table 3a‡

**Table S6: Association between intimate partner violence and explanatory factors among males (Adjusted Odds Ratios – Lifetime in 2022)**

|  | **%** | **IPV (All types), lifetime** | **%** | **Psychological IPV, lifetime** | **%** | **Physical IPV, lifetime** | **%** | **Sexual IPV, lifetime** |
| --- | --- | --- | --- | --- | --- | --- | --- | --- |
| **Age** |  |  |  |  |  |  |  |  |
| 15-24 | 8.9 | ref | 8.5 | ref | 9.2 | ref | 20.7 | ref |
| 25-34 | 34.6 | 0.59 (0.37, 0.91)*‡ | 35.6 | 0.69 (0.43, 1.09) | 30.9 | 0.65 (0.26, 1.60) | 37.7 | 0.30 (0.14, 0.66)** |
| 35-44 | 35.3 | 0.62 (0.41, 0.95)* | 35.3 | 0.69 (0.44, 1.08) | 36.4 | 0.73 (0.31, 1.74) | 27.7 | 0.22 (0.09, 0.50)*** |
| 45-54 | 21.3 | 0.50 (0.32, 0.79)** | 20.6 | 0.54 (0.33, 0.87)* | 23.5 | 0.66 (0.25, 1.70) | 14.0 | 0.16 (0.06, 0.42)*** |
| **Place of residence** | | |  |  |  |  |  |  |
| Urban | 41.6 | ref | 41.3 | ref | 40.5 | ref | 48.2 | ref |
| Rural | 58.4 | 0.78 (0.61, 0.998)* | 58.7 | 0.82 (0.63, 1.05) | 59.5 | 0.79 (0.54, 1.15) | 51.8 | 0.69 (0.44, 1.09) |
| **Education level** | | |  |  |  |  |  |  |
| No education | 2.7 | ref | 2.7 | ref | 2.3 | ref | 2.2 | ref |
| Primary | 47.6 | 1.73 (1.02, 2.93)* | 47.8 | 1.73 (0.992, 3.00)‡ | 51.2 | 1.93 (0.86, 4.35)‡ | 44.1 | 1.71 (0.69, 4.24)‡ |
| Secondary | 32.0 | 1.73 (0.988, 3.01)‡ | 31.1 | 1.64 (0.92, 2.90)‡ | 33.3 | 2.12 (0.92, 4.89)‡ | 37.6 | 1.95 (0.74, 5.11) |
| Higher | 17.7 | 1.24 (0.70, 2.20)‡ | 18.4 | 1.31 (0.71, 2.41)‡ | 13.2 | 1.20 (0.47, 3.10) | 16.1 | 1.11 (0.39, 3.18) |
| **Perpetrated physical violence against partner** | | | | |  |  |  |  |
| No | 53.4 | ref | 54.2 | ref | 37.6 | ref | 40.8 | ref |
| Yes | 46.6 | 4.27 (3.39, 5.41)*** | 45.9 | 3.71 (2.90, 4.74)*** | 62.4 | 4.85 (3.24, 7.27)*** | 59.2 | 4.45 (2.68, 7.40)*** |
| **Father beat mother** | | |  |  |  |  |  |  |
| No | 45.4 | ref | 45.7 | ref | 30.1 | ref | 42.4 | ref |
| Yes | 48.7 | 1.14 (0.90, 1.45)‡ | 48.4 | 1.13 (0.89, 1.44) | 65.3 | 2.09 (1.43, 3.06)*** | 55.9 | 1.28 (0.78, 2.11)‡ |
| Don't know | 5.9 | 1.03 (0.68, 1.57) | 5.9 | 1.03 (0.67, 1.59) | 4.6 | 1.15 (0.44, 2.95) | 1.7 | 0.30 (0.10, 0.91)*‡ |

Significance ***p<0.001, **p<0.01, *p<0.05

Change in association compared to Table 3a ‡

**Table S7: Association between intimate partner violence and explanatory factors among males (Adjusted Odds Ratios – 12 months in 2014)**

|  | **%** | **IPV (All types), past 12 months** | **%** | **Psychological IPV, past 12 months** | **%** | **Physical IPV, past 12 months** | **%** | **Sexual IPV, past 12 months** |
| --- | --- | --- | --- | --- | --- | --- | --- | --- |
| **Age** |  |  |  |  |  |  |  |  |
| 15-24 | 8.0 | ref | 7.2 | ref | 12.2 | ref | 7.4 | ref |
| 25-34 | 44.7 | 0.81 (0.44, 1.48)‡ | 43.5 | 0.89 (0.45, 1.77) | 41.3 | 0.49 (0.21, 1.13) | 57.9 | 1.26 (0.52, 3.02) |
| 35-44 | 32.0 | 0.65 (0.35, 1.21)‡ | 32.6 | 0.78 (0.39, 1.56)‡ | 28.7 | 0.40 (0.17, 0.93)* | 23.1 | 0.56 (0.22, 1.53)‡ |
| 45-54 | 15.3 | 0.38 (0.19, 0.73)** | 16.6 | 0.49 (0.23, 1.04)‡ | 17.8 | 0.31 (0.13, 0.77)* | 11.6 | 0.38 (0.15, 0.94)* |
| **Place of residence** |  |  |  |  |  |  |  |  |
| Urban | 43.0 | ref | 41.5 | ref | 45.8 | ref | 50.0 | ref |
| Rural | 57.0 | 1.11 (0.84, 1.48) | 58.5 | 1.20 (0.87, 1.65) | 54.2 | 0.97 (0.64, 1.49) | 50.0 | 0.78 (0.44, 1.39)‡ |
| **Education level** |  |  |  |  |  |  |  |  |
| No education | 1.8 | ref | 2.1 | ref | 1.7 | ref | 0.9 | ref |
| Primary | 53.6 | 2.48 (1.26, 4.88)** | 52.9 | 2.04 (1.03, 4.06)* | 48.8 | 1.99 (0.51, 7.76) | 53.7 | 4.15 (0.83, 20.68)‡ |
| Secondary | 29.2 | 2.64 (1.31, 5.30)** | 28.7 | 2.16 (1.06, 4.42)* | 34.3 | 2.74 (0.68, 10.97)‡ | 30.0 | 4.25 (0.82, 22.13)‡ |
| Higher | 15.4 | 3.47 (1.65, 7.32)** | 16.4 | 3.13 (1.45, 6.74)** | 15.2 | 3.05 (0.70, 13.35) | 15.4 | 4.90 (0.90, 26.58) |
| **Perpetrated physical violence against partner** |  |  |  |  |  |  |  |  |
| No | 38.2 | ref | 37.6 | ref | 32.7 | ref | 37.6 | ref |
| Yes | 61.8 | 3.40 (2.56, 4.50)*** | 62.4 | 3.38 (2.48, 4.60)*** | 67.4 | 3.55 (2.19, 5.76)*** | 62.5 | 2.81 (1.64, 4.84)*** |
| **Father beat mother** |  |  |  |  |  |  |  |  |
| No | 34.6 | ref | 36.3 | ref | 29.3 | ref | 26.2 | ref |
| Yes | 60.6 | 1.64 (1.21, 2.22)** | 58.8 | 1.43 (1.05, 1.93)* | 64.5 | 1.88 (1.13, 3.13)* | 68.6 | 2.40 (1.25, 4.63)** |
| Don't know | 4.8 | 0.93 (0.55, 1.57) | 4.9 | 0.89 (0.51, 1.57) | 6.3 | 1.47 (0.56, 3.76) | 5.3 | 1.44 (0.41, 5.12) |

Significance ***p<0.001, **p<0.01, *p<0.05

Change in association compared to Table 3b ‡

**Table S8: Association between intimate partner violence and explanatory factors among males (Adjusted Odds Ratios – 12 months in 2022)**

|  | **%** | **IPV (All types), past 12 months** | **%** | **Psychological IPV, past 12 months** | **%** | **Physical IPV, past 12 months** | **%** | **Sexual IPV, past 12 months** |
| --- | --- | --- | --- | --- | --- | --- | --- | --- |
| **Age** |  |  |  |  |  |  |  |  |
| 15-24 | 11.6 | ref | 11.3 | ref | 10.6 | ref | 24.0 | ref |
| 25-34 | 38.7 | 0.51 (0.33, 0.81)** | 38.8 | 0.55 (0.34, 0.91)*‡ | 35.5 | 0.66 (0.22, 1.98) | 47.2 | 0.34 (0.14, 0.82)*‡ |
| 35-44 | 31.2 | 0.39 (0.25, 0.62)*** | 32.1 | 0.43 (0.26, 0.72)** | 31.8 | 0.55 (0.19, 1.57)‡ | 23.6 | 0.17 (0.06, 0.47)** |
| 45-54 | 18.5 | 0.32 (0.20, 0.52)*** | 17.8 | 0.33 (0.20, 0.56)*** | 22.2 | 0.54 (0.17, 1.76)‡ | 5.2 | 0.05 (0.02, 0.16)*** |
| **Place of residence** |  |  |  |  |  |  |  |  |
| Urban | 41.9 | ref | 41.2 | ref | 43.7 | ref | 58.0 | ref |
| Rural | 58.1 | 0.85 (0.67, 1.11) | 58.8 | 0.88 (0.67, 1.15) | 56.3 | 0.70 (0.44, 1.12) | 42.0 | 0.50 (0.30, 0.85)* |
| **Education level** |  |  |  |  |  |  |  |  |
| No education | 2.6 | ref | 2.7 | ref | 2.3 | ref | 1.5 | ref |
| Primary | 46.3 | 1.69 (0.91, 3.14)‡ | 47.5 | 1.67 (0.87, 3.23)‡ | 50.6 | 1.93 (0.71, 5.27) | 39.4 | 2.44 (0.64, 7.91)‡ |
| Secondary | 32.3 | 1.68 (0.86, 3.29)‡ | 30.6 | 1.52 (0.76, 3.04)‡ | 35.2 | 2.08 (0.74, 5.89)‡ | 41.5 | 2.66 (0.71, 9.96)‡ |
| Higher | 18.9 | 1.33 (0.67, 2.66)‡ | 19.2 | 1.32 (0.63, 2.76)‡ | 11.9 | 1.01 (0.30, 3.40) | 17.6 | 1.49 (0.36, 6.11) |
| **Perpetrated physical violence against partner** |  |  |  |  |  |  |  |  |
| No | 54.4 | ref | 55.3 | ref | 37.7 | ref | 41.8 | ref |
| Yes | 45.6 | 3.17 (2.45, 4.11)*** | 44.7 | 2.86 (2.16, 3.79)*** | 62.3 | 4.53 (2.71, 7.59)*** | 58.2 | 4.19 (2.23, 7.86)*** |
| **Father beat mother** |  |  |  |  |  |  |  |  |
| No | 46.5 | ref | 46.2 | ref | 29.6 | ref | 44.4 | ref |
| Yes | 48.4 | 1.12 (0.85, 1.49)‡ | 48.9 | 1.15 (0.87, 1.54)‡ | 64.8 | 2.09 (1.31, 3.32)** | 53.2 | 1.29 (0.68, 2.48)‡ |
| Don't know | 5.1 | 0.86 (0.50, 1.50) | 4.9 | 0.84 (0.46, 1.53) | 5.6 | 1.43 (0.45, 4.55) | 2.4 | 0.48 (0.15, 1.49) |

Significance ***p<0.001, **p<0.01, *p<0.05

Change in association compared to Table 3b ‡
